# Augmenting Endogenous Wnt Signaling Improves Skin Wound Healing

**DOI:** 10.1371/journal.pone.0076883

**Published:** 2013-10-18

**Authors:** Jemima L. Whyte, Andrew A. Smith, Bo Liu, Wilfred R. Manzano, Nick D. Evans, Girija R. Dhamdhere, Mark Y. Fang, Howard Y. Chang, Anthony E. Oro, Jill A. Helms

**Affiliations:** 1 Division of Plastic and Reconstructive Surgery, Department of Surgery, Stanford School of Medicine, Stanford University, Stanford, California, United States of America; 2 Department of Dermatology, Stanford School of Medicine, Stanford, California, United States of America; 3 Howard Hughes Medical Institute, Stanford, California, United States of America; University of Texas Medical Branch, United States of America

## Abstract

Wnt signaling is required for both the development and homeostasis of the skin, yet its contribution to skin wound repair remains controversial. By employing *Axin2^LacZ/+^* reporter mice we evaluated the spatial and temporal distribution patterns of Wnt responsive cells, and found that the pattern of Wnt responsiveness varies with the hair cycle, and correlates with wound healing potential. Using *Axin2^LacZ/LacZ^* mice and an ear wound model, we demonstrate that amplified Wnt signaling leads to improved healing. Utilizing a biochemical approach that mimics the amplified Wnt response of *Axin2^LacZ/LacZ^* mice, we show that topical application of liposomal Wnt3a to a non-healing wound enhances endogenous Wnt signaling, and results in better skin wound healing. Given the importance of Wnt signaling in the maintenance and repair of skin, liposomal Wnt3a may have widespread application in clinical practice.

## Introduction

Injury initiates a local reaction designed to stop blood loss, clear debris, and restore normal function to the damaged tissue. It is this latter step, the restoration of biological function, that diverges dramatically among metazoan species. Many animals can restore damaged integument back to its original architecture but humans, especially the elderly, respond to cutaneous damage in a much more limited fashion. Human skin wound sites typically have a disorganized dermal matrix covered by epithelium devoid of hair follicles and sebaceous glands. When re-epithelialization occurs without epidermal appendage development, skin loses its thermoregulatory and sensory functions and the result is scarring (reviewed in [1]).

Wnt signals play decisive roles in skin development and homeostasis and a key resource in demonstrating these functions has been the generation of Wnt “reporter” mice. The first lines, TOPgal [2] and BATgal [3], exhibit robust activity during development [3]–[5] but are not active in adult skin, or after skin wounding [2]. Because both lines were generated by random insertion they also share risks common to most transgenic lines, including inconsistent reporter activity from generation to generation [6], [7]. Consequently, it has been difficult to define clear role(s) for Wnt signaling in adult skin.

Several lines of evidence indirectly support a role for Wnt signaling in cutaneous repair. For example, Wnt signaling regulates cell proliferation in the adult epidermis [8], which directly impacts the rate and extent of skin wound healing [9], [10]. Wnts also serve as niche signals for at least two types of skin stem cells, those in the bulge region of the hair follicle [4], [11], and those in the basal layer of the interfollicular epidermis [12] and these stem cells contribute to cutaneous wound repair [13]–[15]. The role of Wnt signaling in wound healing, however, remains controversial. For example, inhibition of the Wnt pathway by ectopic expression of Dkk-1 does not retard skin wound healing, but overexpression of Wnt7a promotes the induction of new hair follicles within the wound bed [14], which is typically referred to as “scarless” healing [16]. Here, we examine the temporal and spatial distribution of Wnt responsiveness within the intact skin and during cutaneous healing. We then use an ear wound model to demonstrate how amplifying the Wnt signal in a wound enhances cutaneous repair.

## Results

### Adult Skin is Wnt Responsive

TOPgal and BATgal reporters show weak activity in adult skin; therefore, we used another Wnt reporter strain generated by targeted insertion of the *LacZ* gene into the Axin2 locus [17]. *Axin2* is a direct downstream target of the Wnt pathway [18]; consequently, *LacZ* and its gene product, beta galactosidase, reflect Wnt responsiveness in adult skin.

**Figure 1:**
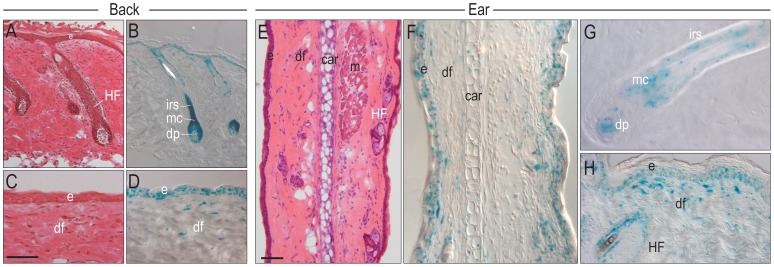
Wnt signaling in back skin compared to the ear. (A) H&E staining of back skin, illustrating hair follicles. (B) Adjacent section from an *Axin2^LacZ/+^* mouse, stained with X-Gal. (C) H&E staining of the epidermis and dermis, and (D) corresponding X-Gal staining. (E) H&E staining of the ear. (F) Adjacent *Axin2^LacZ/+^* section stained with X-Gal. (G) higher magnification of a hair follicle in the ear. (H) X-Gal staining of the epidermis and dermis of the ear. Abbreviations: e, epidermis; df, dermal fibroblasts; HF, hair follicle; dp, dermal papilla; car, cartilage; mc, matrix cells; hs, hair shaft. Scale bars = 50 µm.

We visualized the pattern of Wnt responsiveness in adult skin (Fig. 1). Our analyses began with skin covering the back, where numerous investigators have described the distribution of Wnt-responsive cells in the hair follicle (Fig. 1A). We found that the pattern of X-Gal staining in *Axin2^LacZ/+^* mice accurately reflected Wnt-responsive cells in the hair follicle’s dermal papilla, matrix cells, and the inner root sheath (Fig. 1B). As described [19], we also identified Wnt-responsive cells in the dermis (Fig. 1C,D). The X-Gal staining pattern in the back of *Axin2^LacZ/+^* mice is in keeping with the homeostatic function of Wnt signaling in adult skin [14], [20]–[23].

We evaluated the pattern and level of Wnt responsiveness in other anatomic locations (SFig. 1), including the ear. Skin covering the ear is similar to skin covering the back: both have a surface epidermis with hair follicles and sebaceous glands and a dermis; a thin adipose layer, the panniculus adiposus; and a layer of musculature, the panniculus carnosus (Fig. 1E). The most notable anatomic difference between back and ear skin is the presence of the auricular cartilage (Fig. 1E). Despite this unique anatomical feature (which became beneficial when creating a wound), the distribution of X-Gal^+ve^ cells was similar (Fig. 1F). In the ear we identified Wnt-responsive cells in the dermal papilla, the matrix cells, the inner root sheath of the hair follicle, as well as a population of Wnt-responsive dermal fibroblasts (Fig. 1F–H). Thus, the pattern of Wnt responsiveness in skin and its epithelial appendages is similar, despite regional variations in cutaneous architecture.

### Wnt Signaling Varies with the Hair Cycle

**Figure 2:**
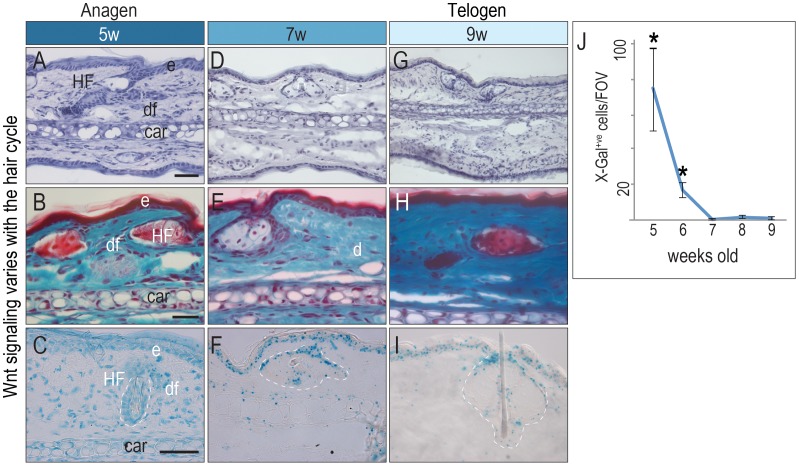
Wnt signaling varies with the hair cycle. (A) Hemotoxylin staining of 5-week-old ear skin. (B) Gomori trichrome staining of 5-week-old ear skin; anagen-stage hair follicles appear red. (C) X-Gal staining of adjacent tissue from a 5-week-old *Axin2^LacZ/+^* mouse. (D) Hemotoxylin staining, (E) Gomori trichrome staining, and (F) X-Gal staining of ear skin from a 7-week-old *Axin2^LacZ/+^* mouse. (G) Hemotoxylin staining, (H) Gomori trichrome staining, and (I) X-Gal staining of ear skin from a 9-week-old *Axin2^LacZ/+^* mouse. (J) The number of Wnt-responsive dermal cells per ROI was quantified at during anagen, catagen, and telogen (N = 3; 3 sections per mouse, 3 mice per week). All mice were male. Abbreviations as in Fig. 1. Scale bars = 50 µm; asterisk denotes P<0.05.

In the hair follicle, BMP signaling is highest during telogen and lowest during anagen and is out of phase with Wnt/beta catenin signaling [20]. We evaluated how the hair cycle influenced the distribution of Wnt responsiveness in the dermis and epidermis. At 5-weeks of age the hair cycle is synchronized [24] and histological analyses confirmed that hair follicles on the ear were in anagen (Fig. 2A,B). The epidermis and hair follicles were sites of high Wnt responsiveness, as was the dermis (Fig. 2C). At 7-weeks of age, histological analyses demonstrated regression of the hair follicles (Fig. 2D,E). The number of X-Gal^+ve^ cells in the dermis was reduced (Fig. 2F). At 9-weeks of age hair follicles are in telogen (Fig. 2G,H) and the number of X-Gal^+ve^ cells in the dermis was reduced further (Fig. 2I). Quantification of the number of X-Gal^+ve^ dermal cells showed that Wnt signaling was highest during anagen, reduced during catagen, and was at its lowest during telogen (Fig. 2J).

### Cutaneous Wound Healing Potential Varies with the Hair Cycle

Wound healing is impacted by the hair cycle [25]. We set out to determine if this was true for wounds at different anatomical locations. We evaluated large wounds created on the back (SFig. 2) and compared them to precise, small injuries created on the ear (SFig. 3A). Although similar results were obtained for wounds in both locales, the ear proved to be a better site for analysis for a number of reasons. First, the auricular cartilage reduces contraction of the wound (SFig. 3A). This is in contrast to back wounds that often rely upon some type of physical constraint to minimize wound contraction (SFig. 3B). Second, the wound size can be standardized and therefore is highly reproducible (SFig. 3C). Furthermore, a circular wound can be generated, which avoids healing differences caused by the orientation of the incision relative to Langer’s lines [26]. The full-thickness ear wound involves the epidermis, dermis, muscle, and extends to the auricular cartilage (SFig. 3D,E). As with other types of wounds, TUNEL staining is evident within 24 h of wounding [27]. We detected TUNEL^+ve^ cells in the epidermis at the wound edge, in dermal fibroblasts, and in the auricular cartilage (SFig. 3F,G). Although the wound edges show evidence of re-epithelialization (SFig. 3I), dermal fibroblasts and chondrocytes near the center of the wound continue to apoptose. In these cases, the wound does not close, which results in a hole (SFig. 3J). This endpoint offers a clear assessment of rapid versus impeded/inadequate healing.

**Figure 3:**
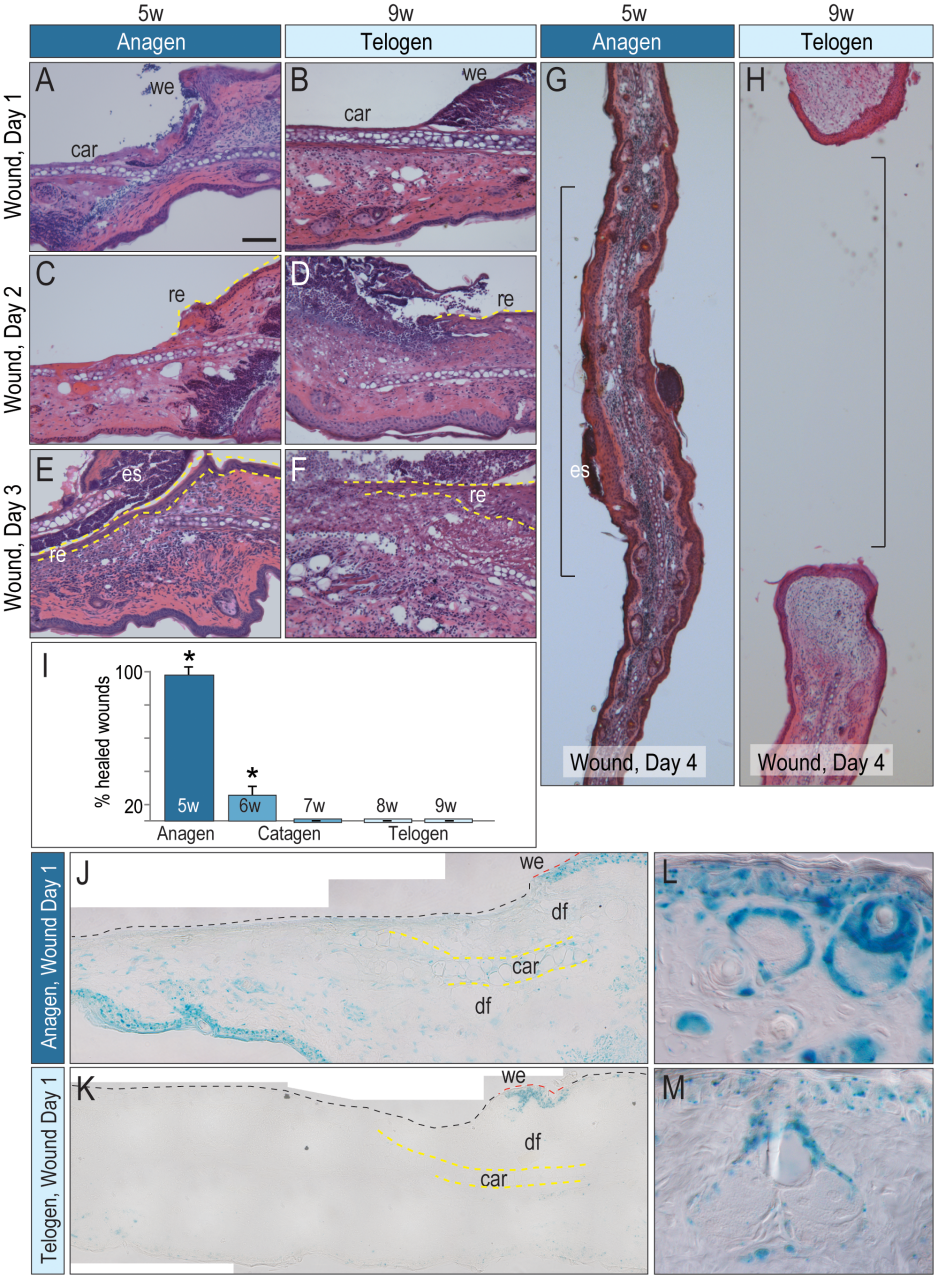
Wound healing and Wnt signaling varies with the hair cycle. On post-wound day 1, H&E staining of injury sites created in (A) anagen or (B) telogen. Post-wound day 2, (C) H&E staining of anagen (D) versus telogen wounds. Dotted yellow line indicates the healing tongue. Post-wound day 3, (E) H&E staining of anagen (F) versus telogen wounds. Dotted yellow line indicates regenerating epithelium. Post-wound day 4, (G) H&E staining of anagen (H) versus telogen wounds. (I) Percentage of healed wounds expressed as a function of the hair cycle (N = 20 per phase). (J) X-Gal staining of a wound bed in anagen versus (K) telogen. Dotted yellow line indicates auricular cartilage. (L) Hair follicles adjacent to the wound edges, in anagen versus (M) telogen. All mice were male. Abbreviations: we, wound edge; re, regenerating epithelium; es, eschar. Scale bar = 50 µm; asterisk denotes P<0.05.

We returned to our question of whether the phase of the hair cycle impacted wound healing. On the first day after wounding, injuries generated during anagen and telogen were indistinguishable (Fig. 3A,B). On day 2, both anagen wounds and telogen wounds showed re-epithelialization at the wound edges (Fig. 3C,D; SFig. 3) but by day 3, anagen wounds were largely re-epithelialized (Fig. 3E) whereas telogen wounds were not (Fig. 3F). By day 4, anagen wounds were fully re-epithelialized (Fig. 3G) but telogen wounds did not finish re-epithelialization and a hole formed (Fig. 3H). We quantified the percent of wounds that healed: 100% of the anagen wounds repaired (N = 20) whereas only 5% of telogen wounds did (N>20; Fig. 3I).

### Wounds Made during Anagen have Elevated Wnt Signaling

The mechanisms responsible for improved wound healing during anagen are not fully understood. We hypothesized that the enhanced wound healing was related to endogenous Wnt signaling, which is at its nexus during anagen (Fig. 2). First, we evaluated the number of Wnt-responsive cells in the anagen and the telogen wounds. X-Gal^+ve^ cells were evident throughout the anagen wound edges, the dermis, and in the auricular cartilage (Fig. 3J). There were far fewer Wnt-responsive cells in telogen wound sites (Fig. 3K), except in the elevated Wnt signaling at the wound edge (labeled “we”). We confirmed that anagen wounds were actually created during anagen (and telogen wounds during telogen) by examining hair follicles at the wound edge and verifying their Wnt responsive status with X-Gal staining (Fig. 3L,M). Thus we conclude that wound-healing potential positively correlates with elevated levels of Wnt signaling. But is it really Wnt activity- or some other factor- that changes with the hair cycle and thus impacts wound healing?

### Amplified Wnt Signaling in Axin2^LacZ/LacZ^ Mice Correlates with Improved Cutaneous Repair

To directly test the role of Wnt signaling in the skin healing process, we turned to a genetic model of enhanced Wnt signaling. In this new series of experiments, all wounds were created in the non-healing, telogen phase using age- and sex-matched controls. *Axin2^LacZ/LacZ^* mice lack both copies of the negative Wnt regulator, Axin2 [28], which results in a ligand-dependent amplification in endogenous Wnt signaling [29]. We began by carefully examining the intact skin of age- and sex-matched *Axin2^LacZ/+^* and *Axin2^LacZ/LacZ^* mice. We detected no obvious differences in the number of hair follicles, the thickness of the epidermis, or the dermis between the two genotypes (SFig. 4A,B). In both genotypes, programmed cell death was limited to the upper-most layer of the intact epidermis (SFig. 4C–F) and the number and distribution of BrdU^+ve^ proliferating cells was also similar (SFig. 4G,H).

We then evaluated whether the amplified Wnt signaling environment of *Axin2^LacZ/LacZ^* mice affected wound healing potential. We controlled for temporal and spatial differences in endogenous Wnt signaling by restricting our analyses to telogen wounds generated in 9-week-old mice. Wounds were profiled over 4 days.

**Figure 4:**
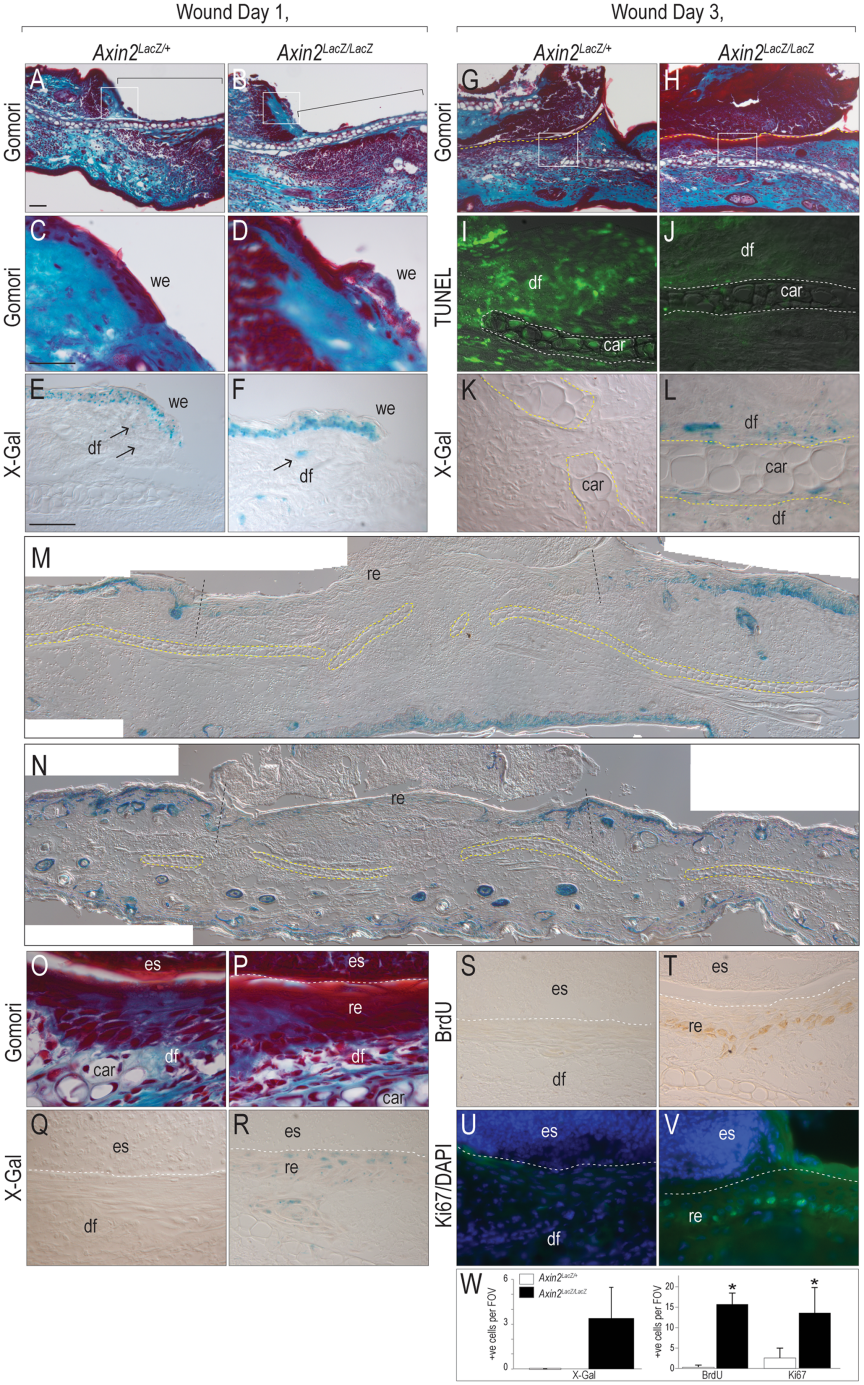
Elevated Wnt signaling in Axin2^LacZ/LacZ^ mice correlates with improved cutaneous repair. (A) Wound on post-injury day 1, stained with Gomori trichrome in *Axin2^LacZ/+^* and (B) *Axin2^LacZ/LacZ^* mice. (C) Wound edge in *Axin2^LacZ/+^* and (D) *Axin2^LacZ/LacZ^* mice. (E) Wound edge stained X-Gal from *Axin2^LacZ/+^* and (F) *Axin2^LacZ/LacZ^* mice. Arrows indicate X-Gal^+ve^ dermal fibroblasts. (G) Wound on day 3, stained with Gomori in *Axin2^LacZ/+^* and (H) *Axin2^LacZ/LacZ^* mice. (I) Wounds stained with TUNEL in *Axin2^LacZ/+^* and (J) *Axin2^LacZ/LacZ^* mice. Dotted line indicates auricular cartilage. (K) X-Gal in *Axin2^LacZ/+^* and (L) *Axin2^LacZ/LacZ^* mice. (M) Wounds on day 3, stained with X-Gal in *Axin2^LacZ/+^* and (N) *Axin2^LacZ/LacZ^* mice. (O) An eschar covers the wound beds in *Axin2^LacZ/+^* mice. (P) Regenerating epithelium covers the wound beds in *Axin2^LacZ/LacZ^* mice. (Q) X-Gal in *Axin2^LacZ/+^* and (R) *Axin2^LacZ/LacZ^* mice. (S) BrdU in *Axin2^LacZ/+^* and (T) *Axin2^LacZ/LacZ^* mice. (U) Ki67 immunostaining in *Axin2^LacZ/+^* and (V) *Axin2^LacZ/LacZ^* mice. (W) Quantification of X-Gal+ve dermal fibroblasts, and proliferating cells in the wound beds of *Axin2^LacZ/+^* and *Axin2^LacZ/LacZ^* mice. All mice were male. Scale bar = 50 µm; asterisk denotes P<0.05.

On day 1, wounds generated in *Axin2^LacZ/+^* and *Axin2^LacZ/LacZ^* mice were indistinguishable (Fig. 4A,B). Both wounds had a healing “tongue” of epidermis (Fig. 4C,D) and in both *Axin2^LacZ/+^* and *Axin2^LacZ/LacZ^* mice, Wnt signaling was evident within that tongue (Fig. 4E,F). We noted, however, that the X-Gal staining intensity was greater in *Axin2^LacZ/LacZ^* mice because each cell expresses two copies of the *LacZ* gene.

We examined the wound beds every 24 h. By day 3, both groups of animals showed evidence of re-epithelialization on the wound edges (Fig. 4G,H). Upon closer examination, however, some important differences were apparent: TUNEL staining was more widespread in *Axin2^LacZ/+^* controls than in *Axin2^LacZ/LacZ^* mice. TUNEL^+ve^ dermal fibroblasts and auricular chondrocytes were detected throughout the *Axin2^LacZ/+^* wound bed (Fig. 4I) and this TUNEL signal was essentially absent in the wound beds of *Axin2^LacZ/LacZ^* mice (Fig. 4J).

The distribution of Wnt-responsive cells in the wound bed was also notably different between *Axin2^LacZ/+^* and *Axin2^LacZ/LacZ^* mice. Auricular chondrocytes and dermal fibroblasts lacked Wnt-responsiveness in *Axin2^LacZ/+^* mice (Fig. 4K), but in *Axin2^LacZ/LacZ^* mice both chondrocytes and dermal cells were Wnt-responsive (Fig. 4L). The absence of Wnt-responsive cells in heterozygote mice was not due to the signal being below the limit of detection: low-magnification images illustrated X-Gal^+ve^ cells in the epidermis, and a notable lack of X-Gal^+ve^ cells in the wound beds of *Axin2^LacZ/+^* mice (Fig. 4M), just as we had observed previously (Fig. 3K). By day 3, wounds created in *Axin2^LacZ/+^* and *Axin2^LacZ/LacZ^* mice both showed breaks in the auricular cartilage (Fig. 4M,N).

We evaluated the wound site. Both *Axin2^LacZ/+^* and *Axin2^LacZ/LacZ^* wound beds have an eschar but only in *Axin2^LacZ/LacZ^* mice was there definitive evidence of re-epithelialization (Fig. 4O,P). In *Axin2^LacZ/LacZ^* mice the epithelial cells were X-Gal^+ve^ (Fig. 4Q,R; quantified in Fig. 4W). BrdU incorporation (Fig. 4S,T) and Ki67 immunostaining (Fig. 4U,V) confirmed that the cells in the *Axin2^LacZ/LacZ^* wound bed were proliferating. By day 4 these differences in Wnt signaling, cell proliferation, and cell death culminated in a divergence in the healing response: As observed previously (Fig. 3), telogen wounds generated in *Axin2^LacZ/+^* mice failed to heal (0/10), whereas telogen wounds generated in *Axin2^LacZ/LacZ^* mice healed (10/10). Thus, genetically amplifying Wnt signaling leads to improved skin wound healing.

### L-Wnt3a Improves Cutaneous Repair

We tested if a Wnt signal was sufficient to rescue telogen wound failures. We generated full-thickness ear wounds in *Axin2^LacZ/+^* mice, and took care to create all wounds during telogen, again using age- and sex-matched animals (Fig. 4). Wounds were treated topically with a lipid formulation of Wnt3a (L-Wnt3a) that maintains the biological activity of the hydrophobic protein [30]. L-Wnt3a was applied to the wound 1×/day and wound responses were evaluated on post-injury days 3 and 4. Control wounds were treated with the same liposomal formulation containing PBS (L-PBS).

**Figure 5:**
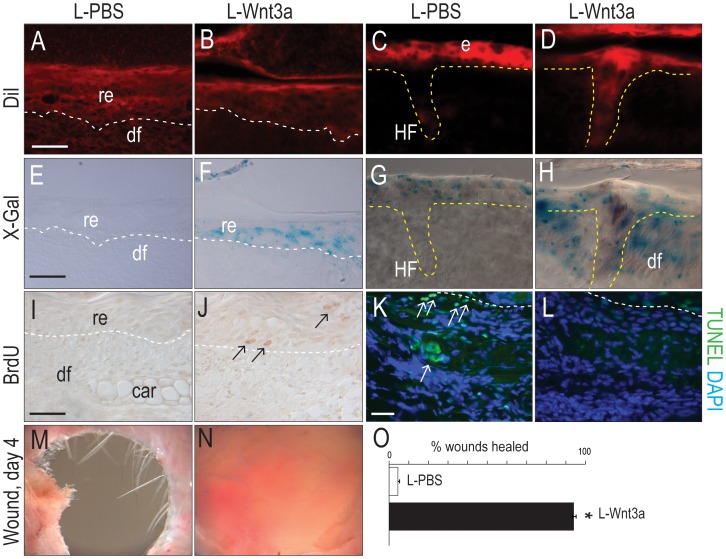
L-Wnt3a stimulates cutaneous repair. (A) Fluorescent signal in the wound bed on day 3 treated with DiI-labeled liposomes containing PBS or (B) Wnt3a. Dotted lines indicate boundary between the epithelium and dermis. (C) Fluorescent signal in hair follicles and epithelium adjacent to the wound bed, treated with DiI-labeled liposomes containing PBS or (D) Wnt3a. (E–H) X-Gal staining of adjacent tissue sections shown in A–D. (I) BrdU staining in the wound bed treated with L-PBS or (J) L-Wnt3a. Arrows indicate immunopositive cells. (K) TUNEL staining in the wound bed treated with L-PBS or (L) L-Wnt3a. Arrows indicate immunopositive cells. (M) Telogen wounds treated with L-PBS fail to heal. (N) Telogen wounds treated with L-Wnt3a heal. (O) Quantification of the percent of L-PBS versus L-Wnt3a treated wounds that healed (N = 10 per treatment). All mice were male. Scale bars = 50 µm; asterisk denotes P<0.05.

First, we tracked the distribution of the liposomes *in vivo* using the lipophilic dye, DiI, which was incorporated into the membrane bilayer of the liposome. A fluorescent signal was detectable in L-PBS and L-Wnt3a treated wound beds and later, in the regenerated epithelium (Fig. 5A,B) as well as in hair shafts at the wound edges (Fig. 5C,D).

Second, we evaluated wound beds for Wnt responsiveness. In wounds treated with L-PBS, X-Gal^+ve^ cells were largely absent (Fig. 5E), which was in keeping with our previous observations of the lack of Wnt signaling in telogen wound beds (e.g., Fig. 3K; also see Fig. 4M,Q). In contrast, telogen wound beds treated with L-Wnt3a were populated with X-Gal^+ve^ cells, particularly in the regenerated epithelium (Fig. 5F). Even on the wound edges, where intact hair follicles resided, Wnt signaling was increased by L-Wnt3a treatment (compare Fig. 5G with H). We also used qRT-PCR to confirm that L-Wnt3a up regulated Wnt signaling in the treated wound. Relative to controls the Wnt targets *Axin2*, *Tcf4* and *Lef1* were increased in L-Wnt3a treated tissues (SFig. 5). Thus, topical L-Wnt3a was effective at increasing Wnt signaling within the wound.

Third, we evaluated the cellular response to enhanced Wnt signaling. Increased Wnt signaling in the wound epithelium was accompanied by an increase in BrdU^+ve^ cells (Fig. 5I,J). Another major difference between wounds treated with L-PBS and those treated with L-Wnt3a was the extent of cell death: in L-PBS treated wounds, TUNEL^+ve^ cells were evident throughout the regenerating epithelium (Fig. 5K); in L-Wnt3a treated wounds, TUNEL staining was almost nonexistent (Fig. 5L).

Finally, we evaluated the extent of healing in L-PBS and L-Wnt3a treated ear wounds. By day 4, L-PBS treated wounds failed to heal, resulting in a hole (Fig. 5M) whereas L-Wnt3a ear skin wounds healed (Fig. 5N; quantification in Fig. 5O). Collectively, these data demonstrate that topical application of L-Wnt3a is sufficient to activate Wnt signaling in the regenerating epithelium, leading to increased cell proliferation and decreased programmed cell death in the wound, which together contributed to a robust healing response.

## Discussion

Skin regeneration- versus scarring- remains the sought-after objective for wound healing research. Scarring has long been thought to represent the “default” state for injured mammalian skin [31]. Now, new data are calling this long-held dogma into question: The African spiny mouse, *Acomys*, is unusually efficient at regenerating large regions of epidermis after injury, complete with hair follicles and sebaceous glands [32]. The mechanism(s) responsible for this remarkable regenerative potential have yet to be identified but they strongly suggest that mammalian skin regeneration is an achievable goal [33], [34].

### Developing a Robust Model of Skin Wound Healing

The goal of all wound-healing models is to recapitulate clinical problems, to be reliable and reproducible, and to have a definitive readout. Skin wound healing models are no exception: Humans exhibit extensive scarring, but similar large injuries created in mice primarily heal through contraction of the wound margins [35]. In order to recapitulate the clinical problem of scarring, investigators have resorted to creating large back wounds in mice that are prevented from contracting by splints and other devices. We opted to use a much smaller wound model in the ear. Besides their overall size, the major difference between back and ear skin is the presence of the auricular cartilage in the latter, which retards wound contraction. This can be viewed as a positive feature because it allows for the generation of reproducibly sized injuries. The wound healing endpoints of re-epithelialization and dermal remodeling remain the same [36]. A dramatic example of the equivalency between back wounds and ear wounds is the recent demonstration that injuries to the dorsum and to the ears of *Acomys kempi* both regenerate to the same remarkable degree [32].

### Wound Healing Potential, and Wnt Signaling, Fluctuates with the Hair Cycle

Using a back wound model, investigators showed that wounds heal faster during anagen [25]. Using an ear wound model, we demonstrate a similar result: full thickness injuries created during anagen completely heal, while those created during telogen do not. A number of explanations have been put forth: keratinocytes derived from adnexal structures are known to contribute to wound healing [13], [37] and during anagen, wounds may re-epithelialize faster because of this increase in keratinocyte proliferation [25]. A reduction in inflammatory cell infiltration has also been described, which may contribute to faster wound healing (reviewed in [38].

We explored a different hypothesis, that endogenous Wnt signaling was a key factor in wound healing potential. Dermal Wnt signaling was highest during anagen and lowest during telogen, which paralleled wound healing potential. In the hair follicle itself, endogenous Wnt signaling is highest during anagen and lowest during telogen [20]. We do not yet know what factors control endogenous Wnt fluctuations in the skin. Even though enormous efforts have gone into understanding the mechanisms of Wnt gene regulation, this remains an unanswered- and very important- question.

### A Genetic Model of Enhanced Wnt Signaling Correlates with Improved Cutaneous Repair

We demonstrated a correlation between anagen, endogenous Wnt signaling, and would healing potential. During anagen, hair follicle stem cells are activated and these cells- or their progeny- may contribute to the improved wound healing observed during anagen [23], [39]. Dermal and epidermal cell proliferation are elevated during anagen, which might also contribute favourably to wound repair. We provided evidence that anagen is also associated with higher levels of endogenous Wnt signaling, and that along with this elevated Wnt signaling we noted robust healing of ear wounds.

To more definitively separate the effects of the hair cycle from the function of Wnt signaling in wound repair we turned to a genetic model of amplified Wnt signaling. In the heterozygous state, *Axin2^LacZ/+^* mice function as Wnt reporters [17], [18] but in the homozygous state, *Axin2^LacZ/LacZ^* mice have amplified endogenous Wnt signaling [29]. Unlike models of constitutively Wnt signaling, however, Wnt signaling is still ligand-dependent in *Axin2^LacZ/LacZ^* mice [29].

Ear wounds were created in *Axin2^LacZ/+^* mice during telogen. As we observed previously in wild-type mice, these telogen wounds did not heal. In sharp contrast, the same telogen wounds created in *Axin2^LacZ/LacZ^* mice healed. There were a number of striking differences between wounds created in *Axin2^LacZ/+^* and *Axin2^LacZ/LacZ^* mice. First, the regenerating epithelium of *Axin2^LacZ/LacZ^* mice was Wnt-responsive. A similar finding was recently reported in *Acomys* mice, where *Lef1* was expressed in the regenerating epithelium of the spiny mouse but was conspicuously absent from the wound epithelium of *Mus musculus* [32]. Second, in the *Axin2^LacZ/LacZ^* wound beds, TUNEL^+ve^ cells were undetectable. The Wnt-responsive status of auricular chondrocytes may also be important for chondrocyte survival: apoptosis is reduced in the Wnt-responsive cartilage, while apoptosis is elevated in cartilage that is not Wnt-responsive.

There is one caveat to the experiments conducted in *Axin2^LacZ/LacZ^* mice. These mice appear to have more stem cells in their adult tissues, and these tissue-resident stem cells may enhance cell proliferation during healing [29], [40]. Also, in *Axin2^LacZ/LacZ^* mice Wnt signaling is amplified in all tissue compartments. Since Wnt signaling from subcutaneous fat contributes to wound healing [41], we cannot pinpoint which Wnt source is responsible for the enhanced skin wound healing in this mouse model.

### Enhancing Skin Wound Healing with Liposomal Wnt3a

We used a liposomal formulation of Wnt3a protein to treat skin wounds created during telogen. In *Axin2^LacZ/+^* mice these telogen ear wounds do not heal. L-Wnt3a activated the endogenous Wnt pathway in the wound bed, predominantly in the regenerating epithelium. This restriction to the epithelium was directly related to the penetration of L-Wnt3a into the tissues, as shown by tagging the liposome with DiI. Over-expressing Wnt genes in the epidermis leads to dermal fibroblast proliferation [42] and hair follicle initiation [43] and our data from L-Wnt3a trials suggests a similar effect: topical application of this stem cell factor reduces programmed cell death, increases epithelial proliferation, and recapitulates the improved healing we observed in anagen wounds and *Axin2^LacZ/LacZ^* wounds. Collectively, these data provide strong evidence that augmenting the body’s endogenous Wnt signal leads to improved cutaneous repair.

## Materials and Methods

### Animals

The Stanford Committee on Animal Research approved all procedures. *Axin2^LacZ/+^* mice were obtained from Jackson Laboratory (Bar Harbor, ME).

### Ear Wound Model

Using a punch biopsy and scalpel, a 2-mm full-thickness skin wound was made on the ventral surface of the ear down to the cartilage; the epidermis and dermis were removed. Vaseline was applied post-wounding to prevent desiccation. BrdU (1 mg/g) was injected IP 90 minutes prior to tissue harvest.

### Histology and Immunohistochemistry

X-Gal staining detects the LacZ product, β-galactosidase. Tissues were fixed with 0.4% PFA (3 h, RT) then immersed in 30% sucrose (4°C). Tissues were embedded in OCT and cryosectioned (10 µm) then fixed with 0.2% gluteraldehyde (15 m) and stained with X-Gal (37°C).

### Histological Staining

Samples were fixed 4.0% PFA, immersed in hematoxylin (30 s) then in Gomori trichrome (10 m), or eosin (10 m) and differentiated in 0.2% acetic acid.

### TUNEL

TUNEL (Roche, Indianapolis, IN) was performed as described by the manufacturer.

### Cell Proliferation

5′-Bromo-2-deoxyuridine (BrdU; Invitrogen) was injected intra-peritoneally 90 minutes prior to tissue harvest. Tissues were fixed in 4% PFA (1 h, 23°C) then passed through a sucrose gradient (12 h, 4°C). BrdU was developed using DAB (Vector laboratories). Regions of interest (ROI) within the wound bed were photographed (minimum of 10 images/sample) and the number of DAB^+ve^ cells were counted.

The distribution of proliferating cells was verified using Ki67 immunostaining on adjacent sections. Compared to BrdU, which is incorporated into DNA during S-phase, the nuclear protein Ki67 is expressed in all phases of the cell cycle [44].

### Imaging

Imaging was performed with Leica MZ16F fluorescent microscope; Adobe Photoshop CS5 and ImageJ software were used to calculate wound area. Ethidium bromide staining of whole tissues was used to capture fine morphological detail by briefly incubating tissues in a dilute solution of Ethidium bromide/PBS then photographed under UV light [45]–[47].

### Liposomal Preparation and Delivery

Liposomal Wnt3a (effective concentration = 0.05 ng/µl) was prepared as described [30]. PBS liposomes acted as control. Each 2-µl dose was topically applied 1×/day. Liposomes were labeled with DiI (Invitrogen) at a 1∶10,000 molar ratio (Dmpc:DiI). DiI, which only fluoresces when incorporated into a liposomal bilayer, was imaged using UV light.

### Quantitative RT-PCR

Tissue was harvested using a 3 mm punch biopsy, homogenized in TRIzol (Invitrogen), RNA quantified, and quantitative RT-PCR performed (Quantace Bioline, Taunton, MA). Expression levels were calculated using the 2∧-(ddCt) method, normalized to GAPDH [48] and converted to fold-expression compared to intact tissues from the *Axin2^LacZ/+^* mice. The following primer sets were used: GAPDH, acccagaagactgtggatgg and ggatgcagggatgatgttct. Axin2, acacatgcagaaatgggtca and ggacgtctgtgacaagcaga. Tcf4, actgctccttaaccccgttt and cttgcgtctgcgattcataa. Lef1 aggagcccaaaagacctcat and cgtgcactcagctacgacat.

### Statistical Analyses

In all quantitation experiments, results are expressed as the mean +-SD. Statistical differences between sets of data were determined by using student t-tests in Microsoft Excel.

## Supporting Information

Figure S1 **Distribution of X-Gal^+ve^ cells in the skin.** In *Axin2^LacZ/+^* mice, (A) Gomori staining of ventral foot skin characterized by a callus, thick epidermis and a lack of hair follicles. (B) X-Gal^+ve^ cells in the epidermal-dermal boundary. (C) Gomori staining of tail skin with large hair follicles and a thin cuticle. (D) X-Gal^+ve^ cells in the hair follicles, the epidermis, and the dermis. Abbreviations: e, epithelium; df, dermal fibroblasts; HF, hair follicles. Scale bars = 50 µm.
(TIF)

Figure S2 **Back wounds heal similar to ear wounds.** Large, full-thickness back wounds were created in the dorsum of *Axin2^LacZ/+^* mice; on day 3 (A) H&E staining of the healing tongue at the wound edge. (B) Adjacent section stained with X-Gal illustrating positive cells in the dermis underlying the healing tongue. (C) BrdU staining indicates proliferating cells in the healing tongue and underlying tissue. Dotted white line indicates wound edge. (D) H&E staining of the wound bed on day 5. (B) Adjacent section stained with X-Gal illustrating positive cells in the dermis. (C) BrdU staining identifies proliferating cells in the regenerating epithelium and in the wound bed. (G) H&E staining of the wound bed on day 10. (H) Adjacent section stained with X-Gal illustrating positive cells in the periphery but not the center (arrows) of the wound bed. (C) BrdU staining identifies proliferating cells in the regenerating epithelium and in the wound bed. Abbreviations: e, epidermis; car, cartilage; df, dermal fibroblasts; re, regenerating epithelium. Dashed lined outline hair follicles.
(TIF)

Figure S3 **Comparison of the ear and back wound models.** (A) Ear wounds immediately after injury. (B) One-centimeter, bilateral back wounds immediately after injury and placement of splints. (C) Two-millimeter ear wounds elicit minimal bleeding and no trauma. (D) Placement of the ear wound, and (E) schematic illustrating tissues injured by the full-thickness wound. (F) TUNEL staining on post-injury day 1 identifies apoptotic cells in the wound edge, the dermis, and the auricular cartilage. Dotted line indicates wound edge. (G) Apoptotic cells in the wound bed and in the adjacent, intact outer layers of the epithelium. (H) TUNEL staining is evident throughout the wound dermis and cartilage on day 3. (I) Ethidium bromide staining of wounds enabled quantification of wound closure of *Axin2^LacZ/+^* over a 4-day period (N = 5). Pixels were quantified using ImageJ. (J) Wounds created during telogen fail to heal, resulting in a hole in the ear by day 4 (N = 20).
(TIF)

Figure S4 **Intact ear skin is equivilant between Axin2^LacZ/+^ and Axin2^LacZ/LacZ^ mice.** (A) Gomori trichrome of intact ear skin from 9-week-old (telogen phase) *Axin2^LacZ/+^* mice. (C) Intact ear skin from *Axin2^LacZ/+^* mice and (D) *Axin2^LacZ/LacZ^* mice stained with TUNEL. Dying cells are limited to the external layers of the epidermis. (E) Adjacent sections from *Axin2^LacZ/+^* mice and (D) *Axin2^LacZ/LacZ^* mice are stained with DAPI. (G) BrdU staining of intact ear skin from *Axin2^LacZ/+^* mice and (H) *Axin2^LacZ/LacZ^* mice shows similar level of cell proliferation in the hair follicles. Abbreviations: e, epidermis; car, cartilage; df, dermal fibroblasts; HF, hair follicles. Scale bar = 50 µm.
(TIF)

Figure S5 **Quantitative RT-PCR of Wnt target genes following L-PBS or L-Wnt3a treatment.** Ear wounds were harvested on day 3 with a 3 mm punch biopsy; RNA was extracted from the tissues and qRT-PCR was performed for Wnt target genes *Axin2*, *Lef1*, and *Tcf4*. qRT-PCR samples were run in triplicate and normalized to GAPDH (N = 4). Asterisk indicates p<0.05.
(TIF)
