# Supplementary figures and images for: Augmenting Endogenous Wnt Signaling Improves Skin Wound Healing
Source: PLoS One. 2013 Oct 18;8(10):e76883. doi: 10.1371/journal.pone.0076883 (PMC3799989; doi:10.1371/journal.pone.0076883)

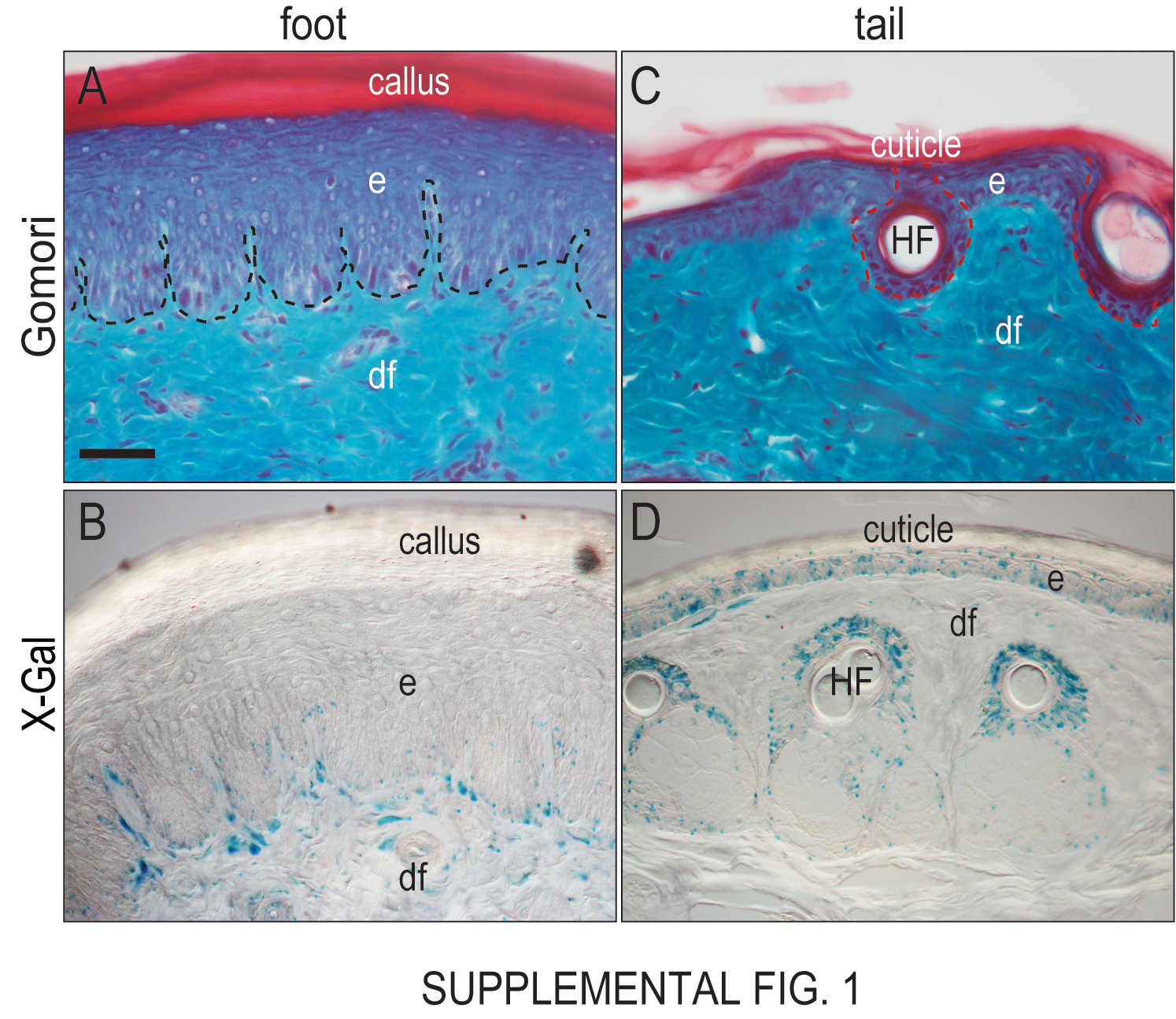

Supplement: Figure S1 — Distribution of X-Gal+ve cells in the skin. In Axin2LacZ/+ mice, (A) Gomori staining of ventral foot skin characterized by a callus, thick epidermis and a lack of hair follicles. (B) X-Gal+ve cells in the epidermal-dermal boundary. (C) Gomori staining of tail skin with large hair follicles and a thin cuticle. (D) X-Gal+ve cells in the hair follicles, the epidermis, and the dermis. Abbreviations: e, epithelium; df, dermal fibroblasts; HF, hair follicles. Scale bars = 50 µm. (TIF) [file pone.0076883.s001.tif]

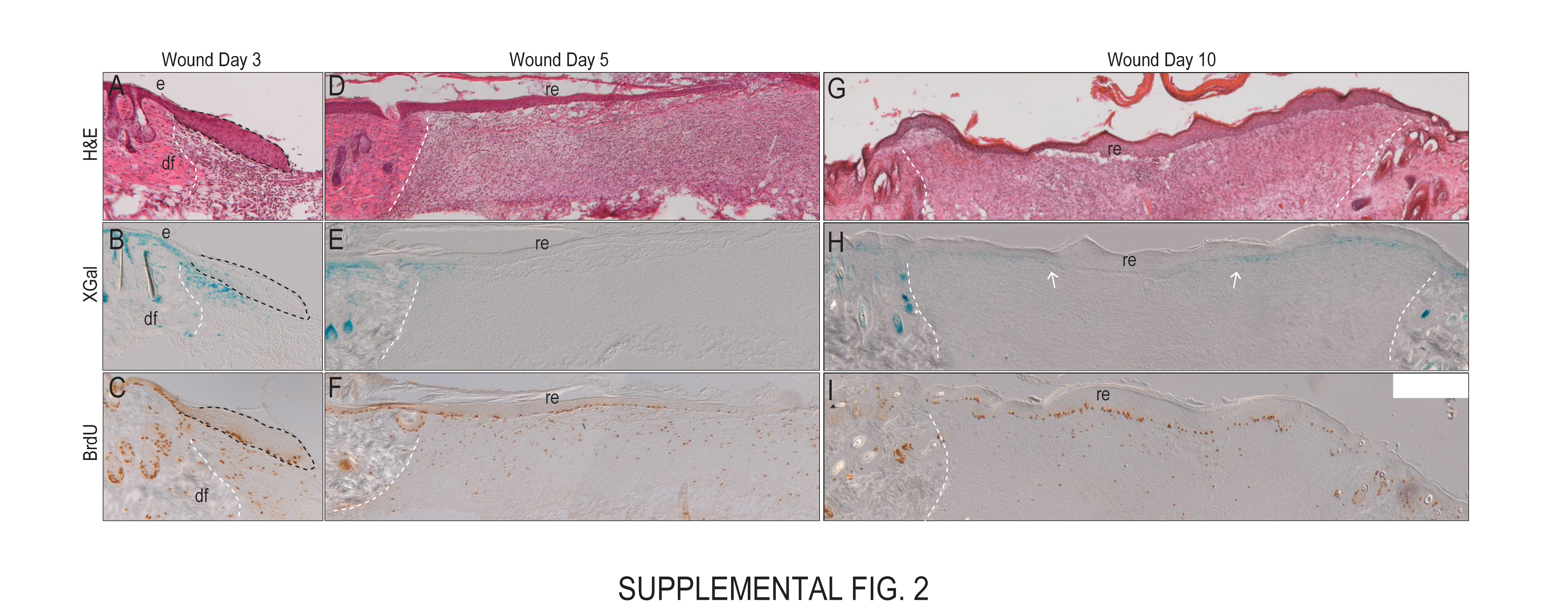

Supplement: Figure S2 — Back wounds heal similar to ear wounds. Large, full-thickness back wounds were created in the dorsum of Axin2LacZ/+ mice; on day 3 (A) H&E staining of the healing tongue at the wound edge. (B) Adjacent section stained with X-Gal illustrating positive cells in the dermis underlying the healing tongue. (C) BrdU staining indicates proliferating cells in the healing tongue and underlying tissue. Dotted white line indicates wound edge. (D) H&E staining of the wound bed on day 5. (B) Adjacent section stained with X-Gal illustrating positive cells in the dermis. (C) BrdU staining identifies proliferating cells in the regenerating epithelium and in the wound bed. (G) H&E staining of the wound bed on day 10. (H) Adjacent section stained with X-Gal illustrating positive cells in the periphery but not the center (arrows) of the wound bed. (C) BrdU staining identifies proliferating cells in the regenerating epithelium and in the wound bed. Abbreviations: e, epidermis; car, cartilage; df, dermal fibroblasts; re, regenerating epithelium. Dashed lined outline hair follicles. (TIF) [file pone.0076883.s002.tif]

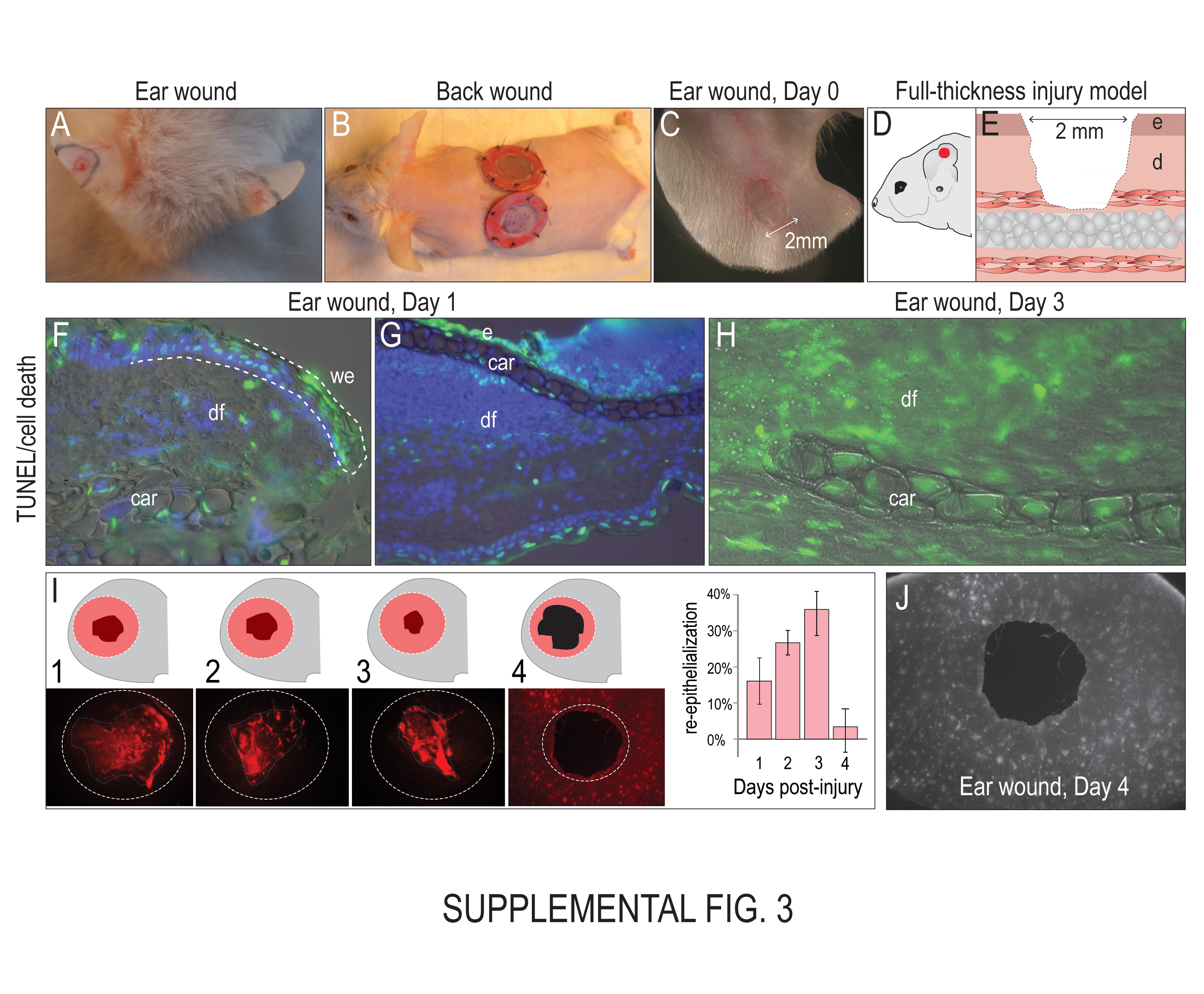

Supplement: Figure S3 — Comparison of the ear and back wound models. (A) Ear wounds immediately after injury. (B) One-centimeter, bilateral back wounds immediately after injury and placement of splints. (C) Two-millimeter ear wounds elicit minimal bleeding and no trauma. (D) Placement of the ear wound, and (E) schematic illustrating tissues injured by the full-thickness wound. (F) TUNEL staining on post-injury day 1 identifies apoptotic cells in the wound edge, the dermis, and the auricular cartilage. Dotted line indicates wound edge. (G) Apoptotic cells in the wound bed and in the adjacent, intact outer layers of the epithelium. (H) TUNEL staining is evident throughout the wound dermis and cartilage on day 3. (I) Ethidium bromide staining of wounds enabled quantification of wound closure of Axin2LacZ/+ over a 4-day period (N = 5). Pixels were quantified using ImageJ. (J) Wounds created during telogen fail to heal, resulting in a hole in the ear by day 4 (N = 20). (TIF) [file pone.0076883.s003.tif]

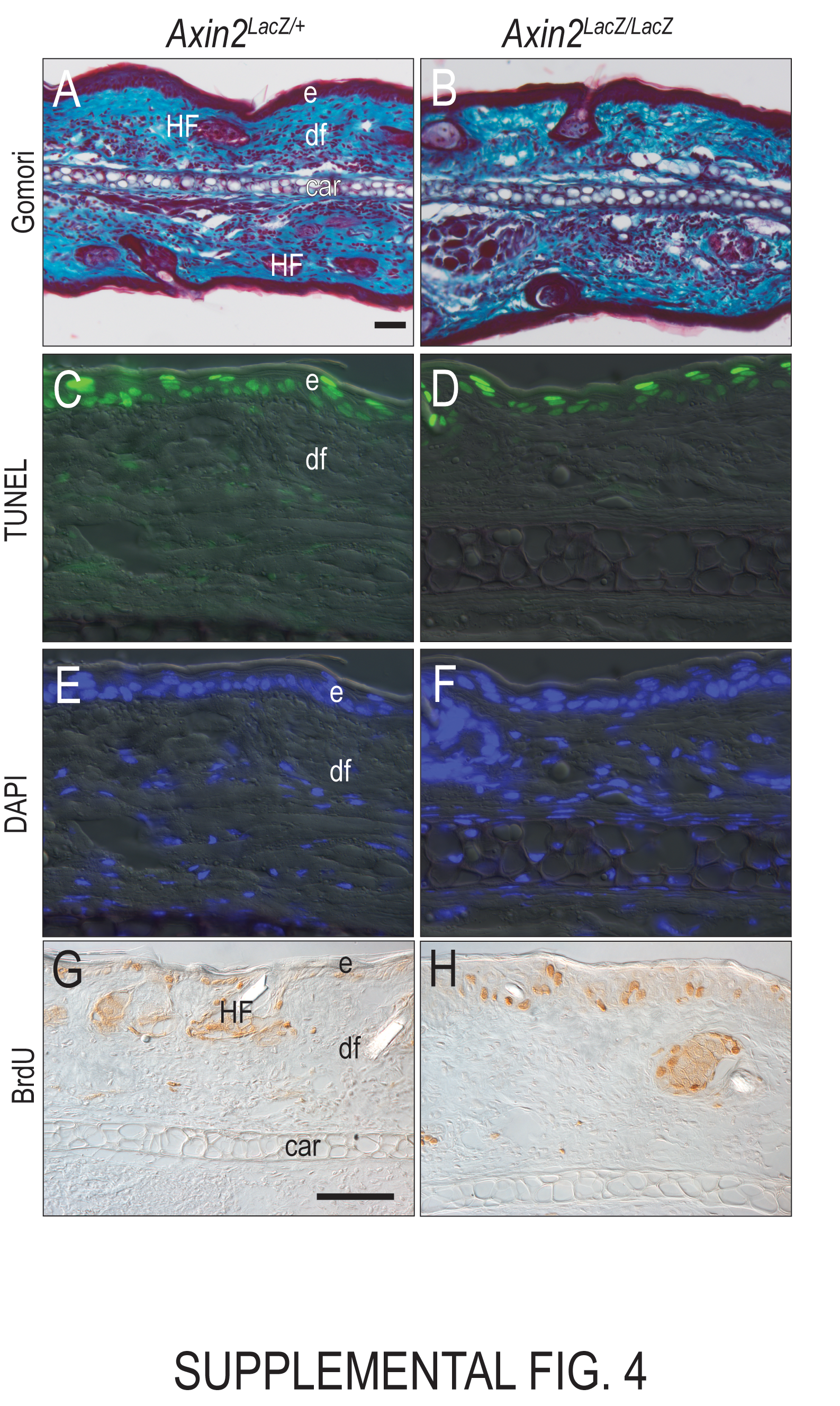

Supplement: Figure S4 — Intact ear skin is equivilant between Axin2LacZ/+ and Axin2LacZ/LacZ mice. (A) Gomori trichrome of intact ear skin from 9-week-old (telogen phase) Axin2LacZ/+ mice. (C) Intact ear skin from Axin2LacZ/+ mice and (D) Axin2LacZ/LacZ mice stained with TUNEL. Dying cells are limited to the external layers of the epidermis. (E) Adjacent sections from Axin2LacZ/+ mice and (D) Axin2LacZ/LacZ mice are stained with DAPI. (G) BrdU staining of intact ear skin from Axin2LacZ/+ mice and (H) Axin2LacZ/LacZ mice shows similar level of cell proliferation in the hair follicles. Abbreviations: e, epidermis; car, cartilage; df, dermal fibroblasts; HF, hair follicles. Scale bar = 50 µm. (TIF) [file pone.0076883.s004.tif]

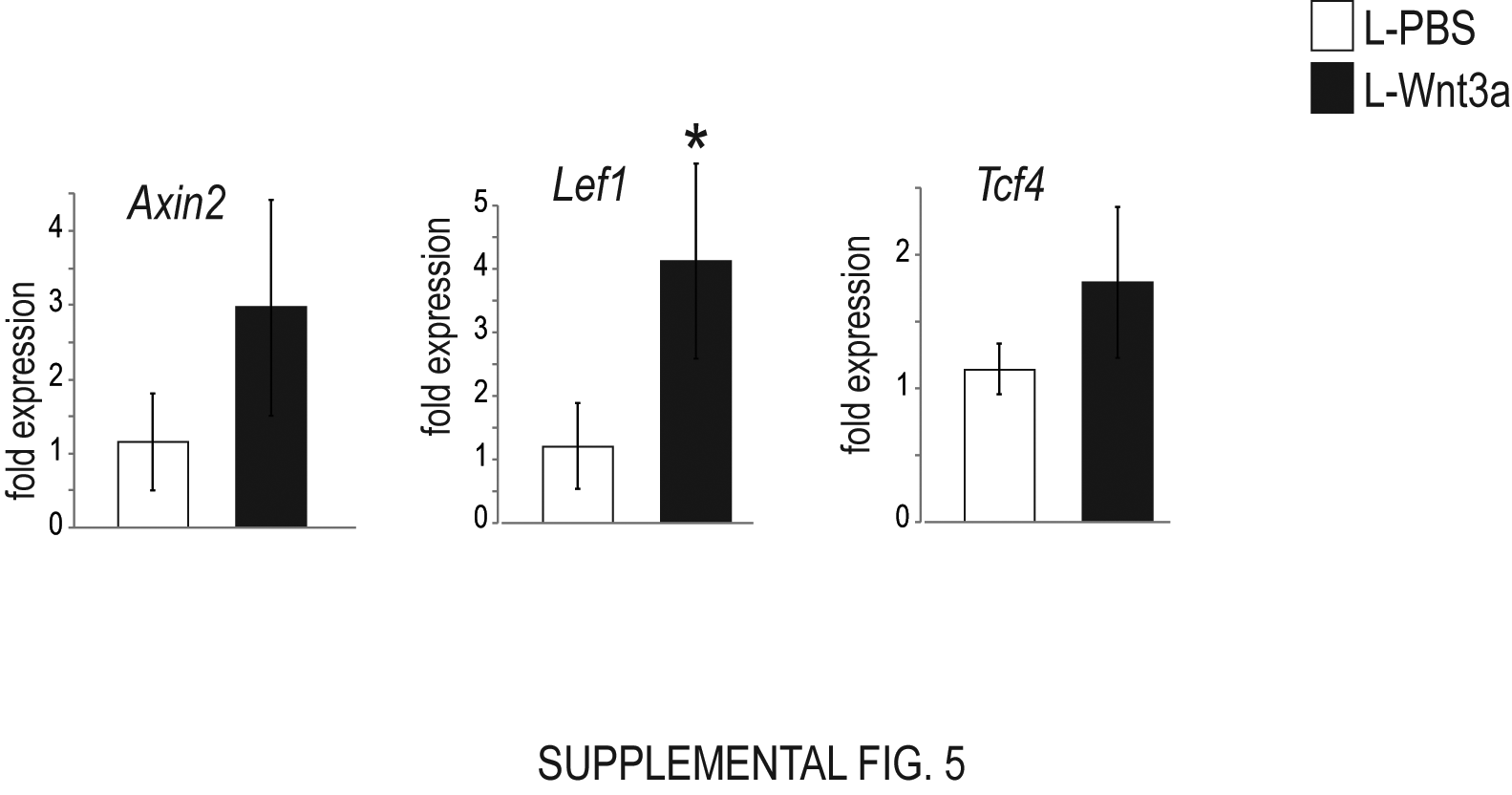

Supplement: Figure S5 — Quantitative RT-PCR of Wnt target genes following L-PBS or L-Wnt3a treatment. Ear wounds were harvested on day 3 with a 3 mm punch biopsy; RNA was extracted from the tissues and qRT-PCR was performed for Wnt target genes Axin2, Lef1, and Tcf4. qRT-PCR samples were run in triplicate and normalized to GAPDH (N = 4). Asterisk indicates p<0.05. (TIF) [file pone.0076883.s005.tif]
